# Odor-Reduced HDPE-Lignin Blends by Use of Processing Additives

**DOI:** 10.3390/polym14132660

**Published:** 2022-06-29

**Authors:** Bianca Lok, Gunnar Mueller, Andrea Buettner, Melanie Bartel, Jens Erdmann

**Affiliations:** 1 Fraunhofer Institute for Process Engineering and Packaging IVV, Giggenhauser Straße 35, 85354 Freising, Germany; andrea.buettner@ivv.fraunhofer.de; 2 Chair of Aroma and Smell Research, Department of Chemistry and Pharmacy, Friedrich-Alexander-Universität Erlangen-Nürnberg, Henkestraße 9, 91054 Erlangen, Germany; 3 Fraunhofer Institute for Applied Polymer Research IAP, Geiselbergstraße 69, 14476 Potsdam-Golm, Germany; gunnar.mueller@prefere.com (G.M.); melanie.bartel@iap.fraunhofer.de (M.B.); jens.erdmann@iap.fraunhofer.de (J.E.)

**Keywords:** activated carbon, bio-based materials, blends, gas chromatography, lignin, olfactometry, plastics, polyolefin, smell, stripping agent

## Abstract

The comprehensive use of natural polymers, such as lignin, can accelerate the replacement of mineral oil-based commodities. Promoting the material recovery of the still underutilized technical lignin, polyolefin-lignin blends are a highly promising approach towards sustainable polymeric materials. However, a limiting factor for high-quality applications is the unpleasant odor of technical lignin and resulting blends. The latter, especially, are a target for potential odor reduction, since heat- and shear-force intense processing can intensify the smell. In the present study, the odor optimization of kraft and soda HDPE-lignin blends was implemented by the in-process application of two different processing additives–5% of activated carbon and 0.7% of a stripping agent. Both additives were added directly within the compounding process executed with a twin screw extruder. The odor properties of the produced blends were assessed systematically by a trained human panel performing sensory evaluations of the odor characteristics. Subsequently, causative odor-active molecules were elucidated by means of GC-O and 2D-GC-MS/O while OEDA gave insights into relative odor potencies of single odorants. Out of 70 different odorants detected in the entirety of the sample material, more than 30 sulfur-containing odorants were present in the kraft HDPE-lignin blend, most of them neo-formed due to high melt temperatures during extrusion, leading to strong *burnt* and *sulfurous* smells. The addition of activated carbon significantly decreased especially these sulfurous compounds, resulting in 48% of overall odor reduction of the kraft blend (mean intensity ratings of 5.2) in comparison to the untreated blend (10.0). The applied stripping agent, an aqueous solution of polymeric, surface-active substances adsorbed onto a PP carrier, was less powerful in reducing neo-formed sulfur odorants, but led to a decrease in odor of 26% in the case of the soda HDPE-lignin blend (7.4). The identification of single odorants on a molecular level further enabled the elucidation of odor reduction trends within single compound classes. The obtained odor reduction strategies not only promote the deodorization of HDPE-lignin blends, but might be additionally helpful for the odor optimization of other natural-fiber based materials.

## 1. Introduction

One of the most common renewable feedstocks is lignin [1], a readily accessible source of sustainable polymeric biomass. Generated during pulping of wooden materials in vast amounts of 50 million tons per year but hitherto often considered as a low-grade by-product, technical lignin is a promising alternative for mineral oil-based commodities [2,3]. It features a complex cross-linked structure on a hetero-aromatic basis [1,4]. For the extraction and isolation of lignin from lignocellulosic material, currently four industrial processes are commonly applied. Divisible into sulfurous and sulfur-free techniques, both the kraft and the sulfite process operate under sulfurous conditions, while the soda and organosolv process yield sulfur-free lignins [5,6,7]. The conditions during the isolation process define not only the purity and the pattern of fragmental products, but the physicochemical properties of the technical lignin in general [8].

Up to now, technical lignin is primarily used as a source of energy; however, the material recovery is on the rise. Apart from the use in duromers and resins, the main field of application proceeds via thermoplastic processing [3,9,10]. Especially as a formulation component in polyolefin composites, technical lignin offers various benefits. Besides increasing the amount of bio-based and sustainable material available at low-cost, such composites can even have superior mechanical properties due to the strengthening effects of the lignin [11,12,13].

The decisive disadvantage, however, is the intense and unpleasant odor of the technical lignin as well as of corresponding polyolefin–lignin blends which limits their acceptance and application possibilities [14]. While disturbing odors emitted by kraft mills or the raw kraft liquor itself are well-known [15,16,17], thorough studies of olfactory detriments of lignin blends are scarce. Due to their dominance in volatile organic compound (VOC) profiles, several phenolic and sulfur compounds were frequently associated with the malodor [5,15,17,18,19]. However, as recently revealed, the olfactory impression of technical lignin and lignin blends comprised a multitude of odor-active substances, partly trace compounds with low odor thresholds [20]. Accordingly, the elucidation of causal odorants at trace level requires the combination of advanced analytics with sensory evaluation.

When it comes to the reduction of odor or VOCs in general, current approaches concentrate on the treatment of the raw materials such as the technical lignin powder. For example, a significant odor reduction was achieved by mixing the lignin powder with activated carbon [21]. Levels of 1−5% by weight of activated carbon proved to be optimal, while no additional odor improvement was observed with higher concentrations. The operating principle of activated carbon is the physical adsorption of organic compounds−among them odorous components–while sorption properties strongly depend on numerous factors such as the shapes of pores, the pore size distribution and the chemical heterogeneity of the surface microstructure [22,23]. However, also chemisorption processes as part of the surface chemistry occur, making the mechanism of adsorption by activated carbons extremely complex [24]. Another approach was the extraction with C_1_−C_4_-alcohols, especially ethanol, which reduced several odorous substances such as dimethyl di-/tri- and tetrasulfide, guaiacol and other phenolic compounds by 50% [25]. Similarly, the enzymatic treatment of technical lignin with different enzymes such as laccases or peroxidases reduced odors moderately [18,26]. Yet, complete deodorization was only achieved by the combination of extraction, enzymatic hydrolysis and extraction with supercritical CO_2_ [14].

The above-mentioned approaches of odor reduction targeted the raw material lignin rather than deodorizing lignin-containing composite materials. However, a high thermal load during the production of such blends leads to additional odor development, potentially even worse with shear-force during extrusion [27,28]. This has already been demonstrated via the detection of additionally formed odorants during extrusion [20]. Likewise, preceding treatment of technical lignin powder led to odor-reduced material, which, however, showed the same level of off-odor after injection molding compared to the untreated lignin [26]. Consequently, focusing on the resulting blend is of prime importance when targeting odor reduction. This has, to date, only been addressed in rare cases regarding wood-based material systems, namely by the addition of zeolites to natural-flour-filled polypropylene [29]. In the case of lignin blends, however, there is a knowledge gap concerning odor reduction.

For this reason, the present study targets the late-stage odor reduction of high-density polyethylene (HDPE)-lignin blends via the addition of two processing additives, being conducted in comparison. Activated carbon and a stripping agent (polymeric, surface-active substances adsorbed onto a PP carrier), respectively, were added during the extrusion, without additional deodorizing processing steps such as extraction or enzymatic treatment. The odor reduction efficiency was assessed by sensory evaluations, and was further substantiated on a molecular level by the identification of odor-active compounds via two-dimensional gas chromatography-mass spectrometry/olfactometry (2D-GC-MS/O). The use both of soda and kraft lignin, in comparison, revealed further insights into effects on these lignins that are produced via different isolation methods. Directly targeting odor reduction within the extrusion process is a novel approach in terms of deodorizing lignin blends. Resulting odor-optimized blends have potential wider fields of application and higher acceptance. Future goal is the replacement of a wide range of conventional HDPE-based products with high-quality lignin blends. Additional benefit is the potential transfer of knowledge and processes for usage within other natural fiber-based materials.

## 2. Materials and Methods

### 2.1. Raw Materials

Besides commercial HDPE for applications in injection molding, also a HDPE-based compatibilizer was also used for the production of the HDPE-lignin blends. For comparison, two different technical lignins were chosen, which had previously proved to be significantly different in odor specifications [20]. One of the chosen lignins resulted from kraft processed softwood, whereas the other lignin was a product of soda processed wheat straw and grass. Both lignins are commercially available and manufactured in high volumes. Prior to compounding, the lignins were dried under vacuum at 60 °C for 24 h.

For potential odor reduction, powdered activated carbon produced by steam activation of selected grades of coal was firstly purified and dried at 280 °C for 4 h according to the manufacturer recommendations and then used for compounding. Another processing additive (‘stripping agent’) was chosen, representing a commercially available solution of polymeric, surface-active substances adsorbed onto a polypropylene (PP) carrier (Table 1). The supplier did not provide any additional information regarding the chemical composition.

### 2.2. Formulation and Preparation of Masterbatches and Blends

Preparation of pre-extrusion mixtures were carried out by means of a Mixer (Turbula Mixer, WAB, Bremerhaven, Germany). The compounding of both the masterbatches and the HDPE-lignin blends was conducted with a twin screw extruder (Leistritz ZSE 18 HP, Leistritz Extrusionstechnik, Nuremberg, Germany) in combination with two dosers (doser A: DDW-MD3-DSR28-20 and doser B: DDW-MD1-MT-10 both with a single spiral screw by Brabender, Duisburg, Germany).

The HDPE-lignin blends were produced with 30% by weight of the respective lignins. Added amounts of the additives were 5% by weight in the case of the activated carbon and 0.7% by weight in the case of the stripping agent. See Table A1 for details in formulation of the masterbatches and the HDPE-lignin blends.

For masterbatch 1, HDPE was mixed with the compatibilizer in a weight ratio of 90:10. Masterbatch 2 additionally included the stripping agent (88.5:9.5:2). After mixing, the respective mixtures were added by means of doser A to the extruder (see Table 2 for technical parameters of extrusion). The resulting polymer strand was led through an ice-cooled water bath and subsequently pelletized by a granulator (SGS 25-E4, Scheer, Germany). Finally, the remaining water was removed from the pellets by drying the material at 40 °C for 16 h in a convection oven.

For the production of both untreated HDPE-lignin blends, masterbatch 1 was added to the extruder barrel by means of doser A, while the kraft and soda lignin, respectively, was added by doser B.

For the blends with activated carbon, the respective lignin was initially mixed with the activated carbon in a weight ratio of 5.8:1 and then added by doser B, while masterbatch 1 was similarly added to the extruder by doser A.

In case of the blends with a stripping agent, both masterbatches were firstly mixed in a 1:1 ratio. The resulting masterbatch mixture was then added to the extruder by means of doser A, while the lignins were applied by doser B, respectively.

The extrusion parameters were comparable for all treated and untreated blends, comprising a melt temperature of 208 °C (Table 2). After extrusion, all HDPE-lignin blends were led through an ice-cooled water bath followed by pelletization and drying at 40 °C.

### 2.3. Sensory Analyses

Five trained panelists (two female and three male) of the Fraunhofer IVV in Freising, Germany, evaluated the sensory characteristics of the sample materials. Their weekly training comprised the recognition of selected odorants out of a large set of common odor-active compounds classified according to an in-house established odor language. Moreover, panel members were tested for normal olfactory function.

The HDPE-lignin blends were randomized and presented to the panel in 140 mL covered glass vessels in two sessions–separated into kraft samples and soda samples. Due to the high odor intensity of the samples, 1 g (±0.1 g) of each blend was sufficient for the determination of sensory characteristics. According to Lok et al. [20], single sensory characteristics were discussed and chosen by the panel in consensus. In this way determined attributes were subsequently rated individually by the panelists on a scale from 0 (no perception) to 10 (strong perception).

The overall odor of the samples was determined comparably by rating the overall odor intensity on a scale from 0−10. In this case, however, the odors of the untreated kraft and soda blends, respectively, were set to 10 and the odor of the respective blends with additives were rated in direct comparison. Hence, ratings were made according to the scale 0 (no perception) to 10 (overall odor of the untreated blends). The significances of differences of mean ratings were calculated by a paired, one- or two-tailed, distributed Student’s *t*-test at α = 0.05.

### 2.4. Olfactometric Instrumental Analyses

#### 2.4.1. GC-O and OEDA

For gas chromatographic analyses, 2 g (±0.1 g) of each HDPE-lignin blend was extracted with 50 mL of dichloromethane (DCM) for 30 min. Filtration, isolation by means of solvent-assisted flavor evaporation (SAFE; [30]) and subsequent concentration via Vigreux and micro distillation [31] were performed with identical work-up as described in Lok et al. [20].

Next, the obtained distillates were diluted stepwise (1:3; *v/v*) with DCM. The resulting 3^n^ series of dilutions corresponded to so-called odor dilution (OD)-factors of 3 to 2187, while the original undiluted distillate referred to as OD-factor 1. This technique–the odor extract dilution analysis (OEDA) [32,33]–serves for the determination of relative odor potencies of single odorants detectable as odor-active compounds during gas chromatographic analyses.

Gas chromatography-olfactometry (GC-O) was performed for the undiluted distillates in triplicate by three assessors with equal instrument as listed in Lok et al. [20]. Separation of volatiles occurred on the capillary column DB-FFAP, whereas odorants were detected simultaneously by means of an odor detection port (ODP) and a flame ionization detector (FID). Likewise, parameters of setting and method have been reported earlier [20]. Analyses of each dilution up to OD-factor 2187 were performed equally.

For the determination of linear retention indices (RIs) of detected odor-active regions, a homologous series of *n*-alkanes (C_6_ to C_26_) was analyzed in parallel [34]. The resultant RIs, together with the odor quality perceived by the assessors at the ODP enabled the tentative identification of odorants. For subsequent unequivocal identification by mass spectral data, gas chromatography-mass spectrometry/olfactometry (GC-MS/O) and two-dimensional (2D-) GC-MS/O were performed and identification criteria were compared to those of reference compounds if available (Section 2.4.2. and Appendix A).

#### 2.4.2. GC-MS/O and 2D-GC-MS/O

GC-MS/O analyses were performed with identical instrument setup and method parameters as previously reported by Lok et al. [20]. Simultaneous to the perception of odor characteristics at the ODP, mass spectra were generated in electron ionization (EI) *full scan* mode over an *m/z* range of 35-399.

Two-dimensional (2D-) GC-MS/O measurements were required for odorous compounds co-eluting with other odorless volatiles, or in the case of ultra-trace odorants below the MS detection limit but perceivable at the ODP. The system consisted of two GCs (7890B GC, Agilent Technologies, Waldbronn, Germany), connected with a cryo-trap system (CTS 1, Gerstel GmbH & Co. KG, Mülheim an der Ruhr, Germany; cooled with liquid nitrogen). Performance of GC-heart-cuts was realized by a multi-column switching system (MCS 2, also Gerstel). This allowed for the transfer of analytes from the first dimension to the second dimension by cryo-trapping (−100 °C), followed by thermodesorption (250 °C). The on-column injection of sample distillates was performed by an autosampler (multipurpose sampler MPS robotic XXL, also Gerstel). The capillary column in the first dimension was a DB-FFAP, while the second dimension was equipped with a DB-5 allowing the separation on two capillary columns of different polarity. Oven temperature programs, details and connections of capillary columns as well as split ratios to the detectors were equal to the previously described system [20]. Detectors in the first dimension were an ODP and FID, whereas the second dimension was equipped with an ODP and MS (5977A single quadrupole MSD, also Agilent; EI, 70 eV over *m/z* 35–400) allowing a simultaneous record of odor quality and mass spectrum (if obtainable).

## 3. Results

### 3.1. Sensory Analysis of Odor Characteristics

**Figure 1:**
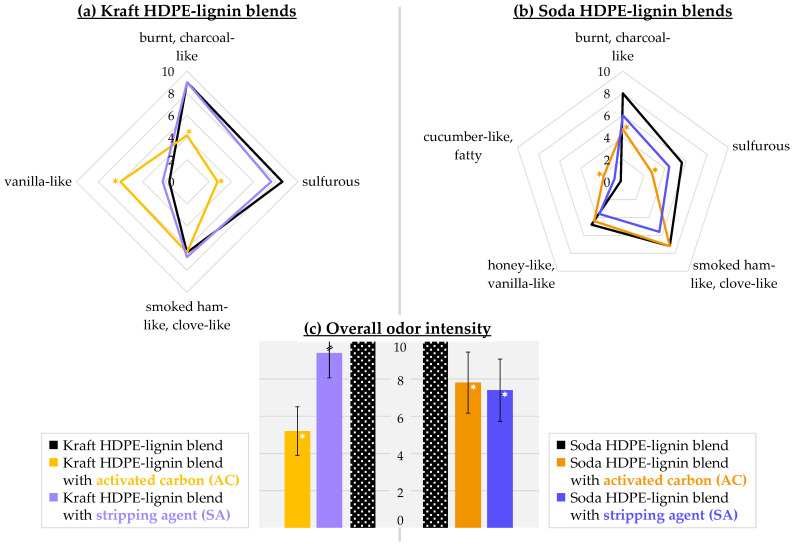
Odor profiles of (**a**) the kraft HDPE-lignin blends and (**b**) the soda HDPE-lignin blends (*n* = 5; scale from 0 (no perception)–10 (strong perception)). (**c**) The overall odor of the kraft and soda blends treated with activated carbon or stripping agent (scale from 0 (no perception)–10 (overall odor of the respective untreated blend)). * significant differences are marked (α = 0.05).

Sensory characteristics of kraft and soda blends were analyzed in separate sessions allowing the exposure of differences in odor between the untreated blend and the odor-reduced blends as precisely as possible. As a first parameter, the overall odor (scale 0–10) of the treated blends were determined in direct comparison to the untreated blends (defined as scale maximum of 10). Second, single odor characteristics were defined session-wise and subsequently rated by the panelists (Figure 1).

#### 3.1.1. Kraft Blends

The overall odor of the kraft blend AC was rated at 5.2, revealing a significant odor reduction of 48% compared to the untreated blend. In contrast, the odor load of the kraft blend SA (intensity rating of 9.4) was as high as the untreated blend. Characterizing the odor in greater detail, the panel defined four odor characteristics: *burnt/charcoal-like*, *sulfurous*, *smoked ham-like/clove-like* and *vanilla-like*. For the kraft blend SA, the mean ratings for all odor impressions were almost identical to the untreated kraft blend with highest ratings for *burnt/charcoal-like* (9.0/9.0) and *sulfurous* (7.6/8.6), followed by *smoked ham-like/clove-like* (6.8/6.4). *Vanilla-like* was perceived with lower intensities (2.2/1.6). The kraft blend AC showed the same *smoked ham-like/clove-like* note as the untreated blend (6.4/6.4), however, significantly lower ratings for *burnt/charcoal-like* (4.2/9.0) and *sulfurous* (2.8/8.6). On the contrary, *vanilla-like* was clearly perceivable in the kraft blend AC only (6.0/1.6).

#### 3.1.2. Soda Blends

With 22% and 26%, respectively, the reductions in the overall odor of the soda blend AC and the soda blend SA were comparable. Next to the odor characteristics *burnt/charcoal-like*, *sulfurous* and *smoked ham-like/clove-like* similarly chosen for the kraft blends, additionally perceptible notes were *honey-like/vanilla-like* and *cucumber-like*. Analogous to the kraft blend SA, the soda blend SA exhibited an odor profile strongly comparable to the untreated blend. However, odor characteristics yielded lower intensities in the soda blend SA compared to the untreated soda blend: 6.0/8.0 for *burnt/charcoal-like*, 4.4/5.6 for *sulfurous*, 5.6/7.2 for *smoked ham-like/clove-like* and 3.6/4.8 for *honey-like/vanilla-like*. Yet, differences in mean ratings were not statistically significant. On the contrary, significantly reduced odor impressions in the case of the soda blend AC were *burnt/charcoal-like* (4.8/8.0) and *sulfurous* (2.8/5.6). *Smoked ham-like/clove-like* (7.2/7.2) and *honey-like/vanilla-like* (4.4/4.8) remained the same, whereas *cucumber-like* was solely perceptible in the soda blend AC (1.8).

### 3.2. Instrumental Analysis of Odorous Compounds

Subsequent gas chromatographic analyses of solvent distillates of all HDPE-lignin blends enabled the elucidation of single odor-active constituents on a molecular level. The determination of odor dilution (OD-) factors reaching from 1 to ≥2187 additionally indicated the odor potencies and the potential extent of contribution of single odorants to the perceived smells.

In the entirety of the sample materials, 70 different odor-active regions were detected during GC-O analyses (Table 3). The identification of causal odorous molecules was successful for 75% by means of (2D-)GC-MS/O, whereby mass spectra were obtainable in 50% of the cases. Hereafter, specific features of odorant patterns are discussed individually for kraft and for soda HDPE-lignin blends.

#### 3.2.1. Odorants in Kraft HDPE-Lignin Blends

The untreated kraft HDPE-lignin blend was the sample with the highest odorant load (56 odorants) closely followed by the kraft blend SA with 52 odorants. In terms of odorants with highest OD-factors, both samples were comparable since 10 and 9 odorants, respectively, were detected with OD-factors 243−2187. The kraft blend AC, however, exhibited substantially fewer odorants: 40 in total, and only 2 corresponding to OD 243. As identified in comparable sample material [20], sulfur compounds (~50%) and differently substituted phenols (~20%) represented the two main substance classes of odorants in kraft HDPE-lignin blends.

##### Sulfur-Containing Odorants

**Figure 2:**
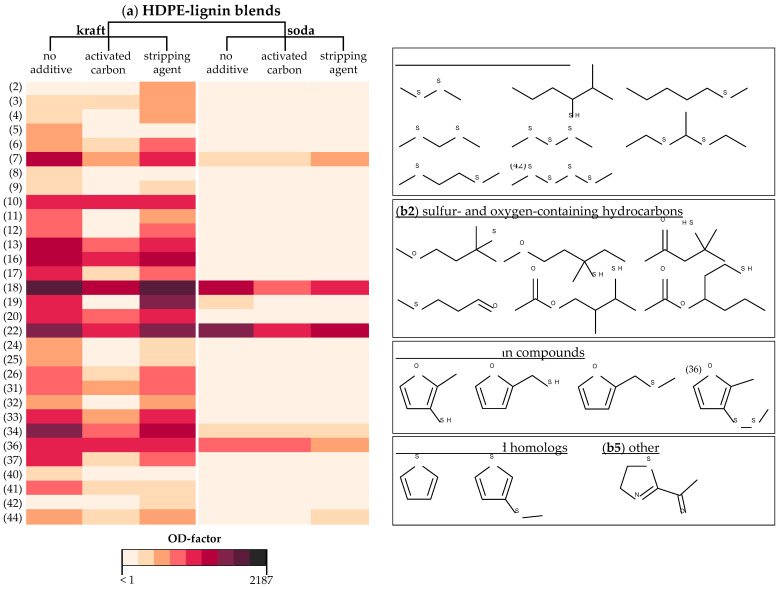
(**a**) Color-illustrated differences in OD-factors of sulfur-containing odorants detected in untreated and treated kraft and soda HDPE-lignin blends and (**b1**–**b5**) structural formulae of sulfur odorants divided into subgroups based on their chemical structure. Numbers (2−44) refer to consecutive order of odorants in Table 3.

The dominating substance class of sulfur compounds included around half of the detected odorants with characteristic *sulfurous*, *burnt*, *garlic-/onion-* or *cabbage-like* smells and was further divisible into four subgroups based on the chemical structure of odorants (Figure 2b). Main odorants of the subgroup of the **sulfur-containing alkanes** (organic sulfides and thiols; No. 5, 6, 7, 13, 18, 20, 25, 42, Table 3) were 1-(methylthio)pentane (No. 7), bis (methylthio)methane (No. 13) and dimethyl trisulfide (No. 18) with OD-factors ≥81 in the untreated kraft blend. **Sulfur- and oxygen-containing hydrocarbons** were mostly detected with OD-factor 27 in the untreated kraft blend, such as 4-methoxy-2-methyl-2-butanethiol (No. 10), 1-methoxy-3-methyl-3-pentanethiol (No. 17), 4-mercapto-4-methyl-2-pentanone (No. 19), 3-mercapto-2-methylbutyl acetate (No. 33) and 1-mercapto-3-hexanyl acetate (No. 37). Out of four detected **sulfurous furan compounds** (No. 16, 22, 26, 36), 2-methyl-3-furanthiol (No. 16) and 2-furfurylthiol (No. 22) showed especially high OD-factors of 81−243. **Thiophene** (No. 3) was less pronounced but its homolog 3-(methylthio)thiophene (No. 34) represented one of the most potent odorants with OD-factor 243 in the untreated kraft blend. Further ten odorants with unknown structure, yet typical *sulfurous* smells were detectable (No. 2, 4, 8, 9, 11, 12, 31, 32, 40, 41).

Out of the 31 sulfur-containing odorants in the untreated kraft blend, only 18 were present in the kraft blend AC. Not only had the number of sulfur compounds decreased, but also the majority was perceived with up to three OD-steps lower (Figure 2). In contrast, most sulfur-containing odorants showed equal OD-factors in the kraft blend SA compared to the untreated kraft blend, whilst only rare cases were detected with one OD-step lower.

##### Phenolic Odorants

Phenols exhibited pronounced OD-factors, above all the *smoky/smoked ham-like* smelling guaiacol and the *vanilla-like* smelling vanillin with the highest OD-factor of ≥2187 in the untreated kraft blend. Further divergently substituted 2-methoxyphenols were isoeugenol (OD-factor 729, No. 65), 2-methoxy-4-vinylphenol (OD-factor 243, No. 61), eugenol (OD-factor 81, No. 59) and with OD-factor 27 2-methoxy-5-methylphenol (No. 51) and 2-methoxy-4-propylphenol (No. 56) with typical *smoky* and/or *clove-like* smells. The role of other phenolic odorants, other than substituted 2-methoxyphenols was negligible since none of them were detected with OD-factors higher than 3.

In the kraft blend AC, the above-mentioned phenolic odorants were generally perceived with 2−3 OD-steps lower. For instance, OD-factors of guaiacol and vanillin decreased from ≥2187 to 243 and also eugenol as well as isoeugenol were perceived with 3 OD-steps lower. In contrast and with a few exceptions only, phenolic odorants were detected with equivalent OD-factors in the kraft blend SA compared to the untreated kraft blend.

##### Minor Compound Classes and Individual Odorants

Apart from sulfur compounds and phenols, only few additional odorants reached high OD-factors; amongst them the *fatty* smelling (*E*)-2-nonenal (No. 28) and the *caramel-like* smelling furaneol (No. 53) with OD-factors of 81 and 243, respectively, in the untreated kraft blend. Both of them featured the same decrease of 2–3 OD-steps in the kraft blend AC but only a single OD-step in the kraft blend SA.

Previously identified in HDPE-lignin blends [20], alkylated 2-cyclopenten-1-ones were similarly detectable in the present samples comprising the *lovage-like* smelling cycloten (No. 46) and the *caramel-like* smelling 3-ethyl-2-hydroxy-2-cyclopenten-1-one (No. 45) and 2-hydroxy-5-ethyl-5-methyl-2-cyclopenten-1-one (No. 47). However, none of them exceeded OD-factor 9. Interestingly, OD-factors were identical in the untreated kraft blend compared to both kraft blends with additives.

#### 3.2.2. Odorants in Soda HDPE-Lignin Blends

Altogether, 41 odorants were detectable in the untreated soda blend, of which 6 had an OD-factor of 243 or higher. The amount and proportion of odorants was lower in the soda blend AC (35 in total, 4 with OD-factor ≥ 243) and even lower in the case of the soda blend SA (32 in total, 2 with OD-factor ≥ 243). Comparable to the kraft blends, phenolic compounds represented the major odorant class whereas sulfur compounds were significantly less pronounced. Additionally, several unsaturated aldehydes were perceivable in the soda blends. Aldehydes, sulfur und phenolic compounds accounted for 60% of all detected odorants in the soda blends.

##### Phenolic Odorants

**Figure 3:**
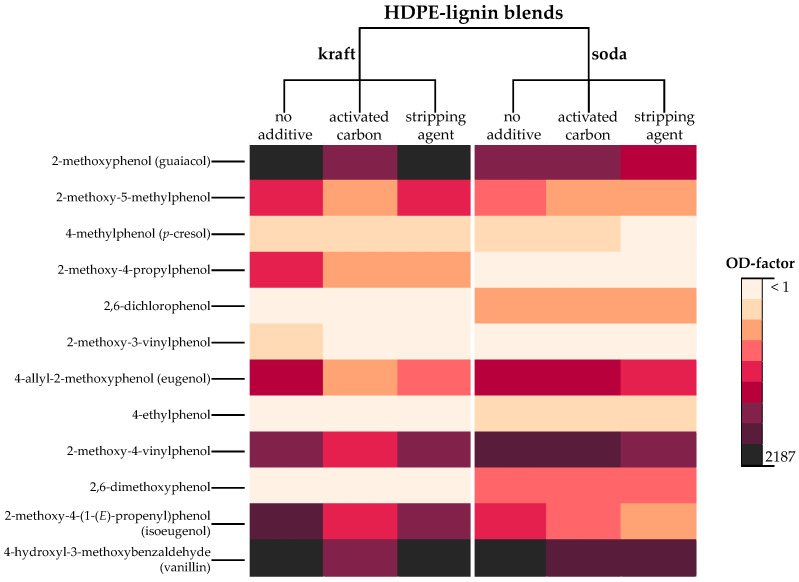
Color-illustrated differences in OD-factors of phenolic odorants detected in untreated and treated kraft and soda HDPE-lignin blends.

Overall, the soda blends were highly comparable to the kraft blends regarding type and load with phenolic odorants (Figure 3). Again, vanillin (No. 69) represented the odorant with the highest OD-factor in the untreated soda blend, followed by isoeugenol (OD-factor 729, No. 65), guaiacol (OD-factor 243, No. 48) and eugenol (OD-factor 81, No. 59). Exclusively perceivable in the soda blends were 4-ethylphenol (*fecal, phenolic*, No. 60) and 2,6-dimethoxyphenol (*smoked ham-like, smoky*, No. 64) but neither of them exceeding OD-factor 9. The pattern of phenolic odorants of the soda blend AC was almost identical to the untreated soda blend. In the case of the soda blend SA however, phenols were homogenously perceived with one OD-step lower.

##### Sulfur-Containing Odorants

With a total of seven sulfur compounds in all three soda blends (No. 7, 18, 19, 22, 34, 36, 44), their load with sulfur-containing odorants was particularly lower compared to the kraft blends (Figure 2). High OD-factors ≥ 81 were only obtained for the *roasted coffee bean-like* smelling 2-furfurylthiol (No. 22) and the *garlic-like, cabbage-like* smelling dimethyl trisulfide (No. 18) in the untreated soda blend. Both odorants were perceived with two OD-steps lower in the soda blend AC and one OD-step lower in the soda blend SA, respectively.

##### Aldehydes

Except for octanal (No. 14) and phenylacetaldehyde (No. 35), the detected aldehydes in the soda blends were mostly unsaturated: (*Z*)-2-nonenal (No. 27), (*E*)-2-nonenal (No. 28), (*E,Z*)-2,6-nonadienal (No. 30), (*E,E*)-2,4-nonadienal (No. 39) and (*E*)-2-undecenal (No. 43). However, only (*E*)-2-nonenal and (*E,Z*)-2,6-nonadienal showed a noteworthy OD-factor of 81 in the untreated soda blend. Interestingly, (*E*)-2-nonenal was detected with comparable OD-factors in the kraft blends, whereas the *cucumber-like* smelling (*E,Z*)-2,6-nonadienal was solely perceived in the soda blends. OD-factors of both of these two unsaturated aldehydes decreased from 81 to 9 in the soda blend AC. The reduction trend was less homogenous in the soda blend SA: one OD-step in case of (*E*)-2-nonenal but three OD-steps in case of (*E,Z*)-2,6-nonadienal.

##### Minor Compound Classes and Individual Odorants

Other than aldehydes, sulfur and phenolic compounds, the *caramel-like* smelling furaneol (No. 53) was detected with OD-factor 243 in the untreated soda blends and OD-factor 27 in both treated soda blends. In contrast to furaneol, that was comparably perceptible in both kraft and soda blends, 3-phenylpropanoic acid was solely perceived in the soda blends. Characterized by a *honey-like* smell, 3-phenylpropanoic acid featured high OD-factors of 81–243 in all soda blends. Alkylated 2-cyclopenten-1-ones were less pronounced in soda blends since not exceeding OD-factor 3.

## 4. Discussion

### 4.1. Comparison of Kraft and Soda HDPE-Lignin Blends and Correlation of Odor Profiles with Main Odorants

The high load of the kraft blends with odorous sulfur compounds can be traced directly to the kraft process using sulfurous reactants for the isolation of lignin. Known to be extremely odor potent [35], the detected sulfur compounds are responsible for the intense *sulfurous* odor impression perceived in kraft blends. Likewise, the combined perception of these sulfurous odorants probably results in the *burnt* odor sensation. Evidence can be found in the case of both AC blends, where the reduction of sulfur compounds led to the decrease of both *sulfurous* and *burnt* odor impressions to a comparable extent. However, as previously described in similar HDPE-lignin blends [20], the detected phenols, furans and alkylated 2-cyclopenten-1-ones might contribute to this *burnt* smell since they have been reported to be contributors in odors of smoke [36,37,38,39,40]. This would explain the same level of perception of the *burnt* note in both untreated soda and kraft blend (8.0/9.0) but lower perception of the *sulfurous* note (5.6/8.6) in the soda blends, since only few sulfur compounds were detected while levels of phenols were comparable.

Due to the absence of sulfurous reactants in the soda process, the *sulfurous* note was less pronounced in the resulting soda blends. Molecularly, almost exclusively dimethyl trisulfide and 2-furfurylthiol were perceivable as sulfurous odorants in the soda blends but both with exceptionally high OD-factors. Therefore, both substances do not indicate sulfurous isolation processes as reported for other sulfur compounds. As known products of thermal processing [41], especially 2-furfurylthiol, they might consequently serve as a general indicator for thermal processing of lignin-containing materials.

Phenols represented the main odorants both in soda and kraft blends to the same extent, comprising the most potent guaiacol and vanillin, the latter being accountable for the perceived *vanilla-like* notes. Guaiacol, however, together with eugenol, isoeugenol and 2-methoxy-4-vinylphenol are known triggers of *smoked ham-like, clove-like* odors, which were perceived with comparable ratios and intensities in all samples. In general, phenolic compounds are common major degradation products of lignin, being derived from its aromatic-based heteropolymer structure [5]. Thus, a wide range of phenolic compounds is not only typical for isolated technical lignin, but has been previously reported in diverse types of wood as natural degradation product of lignin [42,43,44].

The detection of (*E,Z*)-2,6-nonadienal and 3-phenylpropanoic acid in the soda blends only was exceptional, with their *cucumber-like* and *honey-like* notes clearly perceivable by the panel. Apart from that, also furaneol (*caramel-like*) and (*E*)-2-nonenal (*fatty*) were also odorants with high OD-factors in all blends. Their smells, however, were not specifically perceived as individual notes but potentially contributed to other notes, explained by way of additive or synergistic effects [45]; the *caramel-like* note is especially likely to coincide with sweetish impressions like *vanilla-like* or *honey-like*. Unsaturated aldehydes, such as the detected (*E,Z*)-2,6-nonadienal and (*E*)-2-nonenal, are odorous compounds similarly found in different woods, and have been reported to originate from the degradation of fatty acids [43]. Likewise, 3-phenylpropanoic acid was detected in pine-, cedar- and oak wood [42,43,44]. Its sole detection in the soda blends might be related to the specific monomer composition of lignins, which differs between and even within plant species. Typical for grasses and therefore for the soda blends (made out of straw/grass) is the *p*-coumaryl alcohol as monolignol monomer [5], potentially acting as a precursor for the detected 3-phenylpropanoic acid.

### 4.2. Influence of Processing on Odor—Kneader vs. Extruder

The conditions during the blend production, especially the melt temperature, have previously been identified as major factor influencing the odor of HDPE-lignin blends [20]. Allowing a direct comparison, the type of lignin and the lignin weight fraction (30%) in the samples of the present study were identical to the samples previously analyzed by Lok et al. [20]. However, instead of using a kneader (melt temperature of ~195 °C, [20]), the blends were produced by extrusion comprising a higher melt temperature of 208 °C (Table 2).

Previous analyses of the kneaded blends in comparison to the pure lignin powder revealed, that some odorant classes were degraded within the kneading process [20]. This trend was especially pronounced in the case of the aldehydes and alkylated 2-cyclopenten-1-ones. As detected in the present study, those compound classes underwent an even stronger degradation during the extrusion process. For example, hexanal, (*Z*)-4-heptenal, (*E*)-2-octenal and (*E,E*)-2,4-decadienal detected in the kneaded blends, were no longer detected in the extruded samples. Most likely, this was also the reason why the *hay-like* note of the kneaded soda blends was not characteristic anymore for the extruded soda blends.

On the contrary, previous analyses of the kneaded samples revealed an increase of furan and sulfur compounds during the blend production [20]. This led to the assumption that residual sulfur or other non-odorous sulfur compounds acted as odorant precursors, and that the degradation of lignin is defined by the melt temperature which influences the neo-formation of odorants [20]. This appears to be especially valid for the applied melt temperature of 208 °C during extrusion since the thermal degradation of lignin starts at about 200 °C [46]. Furthermore, sulfur is present in lignin in many forms, such as covalently bound sulfur, sulfate ions, elemental sulfur and in adsorbed polysulfide form [47]. The results of the present study support the validity of both assumptions. A much greater amount of sulfur compounds and sulfurous furans was detected in the extruded kraft blends (29 sulfur compounds, thereof 13 with OD-factor ≥ 27) compared to kneaded kraft blends (8 sulfur compounds, thereof 2 with OD-factor ≥ 27, [20]). Additionally, several furan compounds were neo-formed in the extruded kraft blends such as 4-dimethyl-2-pentylfuran and 5-methylfurfural.

In summary, the blend production via extruder with a higher melt temperature of 208 °C clearly led to a severe increase in odor-active sulfur compounds compared to kneaded blends (195 °C). Resulting *sulfurous* and *burnt* odor impressions were hence not only characteristic for kraft blends but also perceivable in extruded soda blends, while being absent in kneaded soda blends. Critical factors for the odor load of HDPE-lignin blends are, therefore, the applied processing conditions and here, above all, the melt temperature. High temperatures favor pyrolytic degradation processes of the lignin not only leading to effects like discoloration, but also to odor formation [27,46]. These effects become even worse with additional shear-force as administered during extrusion [28]. In terms of odor-reduced blends, mild processing conditions are, accordingly, of prime importance.

### 4.3. Odor Reduction Potency of Used Additives

#### 4.3.1. Activated Carbon

The overall odors of both blends treated with activated carbon were significantly reduced. Here, with 48%, the impact was particularly strong for the kraft blend AC compared to 22% in the case of the soda blend AC. Lower *sulfurous* and *burnt* notes were attributable to the reduction of sulfur compounds, being expressed by a lowering of up to three OD-steps. In the case of the kraft blend AC, 30% of sulfur compounds were even no longer perceivable. The significant reduction in sulfur compounds is the main reason for the stronger odor reduction in the kraft blend AC as these substances accounted there for the main part of odorants (half of the detected single odorants) compared to only few sulfur compounds in the soda blend. Potent reduction of sulfur species and sulfurous odorants via activated carbon have been repeatedly reported, especially in terms of fuels or wastewaters [48,49]. As correspondingly demonstrated in the present study, activated carbon proved to be effective in reducing sulfurous odorants and, therefore, especially powerful for the odor reduction of blends produced with kraft lignins.

As a side effect of the reduced *sulfurous/burnt* odor impressions, other smell nuisances were uncovered–such as the *vanilla-like* note in the kraft blend AC and the *cucumber-like* note in the soda blend AC. Evidently, odor reduction with activated carbon not only led to reduced overall odors but also to shifts in odor profiles.

The potential of activated carbon to reduce odor-active compounds was not limited to sulfur compounds. In addition, aldehydes were reduced by up to three OD-steps such as in case of the main potent compounds (*E*)-2-nonenal and (*E,Z*)-2,6-nonadienal with a decrease from OD-factors of 81 to 3 and 9, respectively. Furan compounds were similarly lowered by two OD-steps on average.

The same trend of reduction in OD-factors of two to three steps was observed for the phenolic odorants, however, only in the case of the kraft blend AC (Figure 3). In the soda blend AC, the majority of phenols were detected with the same OD-factor as in the untreated soda blend. A possible explanation is the strong dependence of the adsorption capacity of activated carbon on numerous factors, namely the particle size, porosity, surface microstructure and organic material it is made of [22]. Both ways of adsorption, physisorption and chemisorption [24], are moreover influenced by the characteristics of the adsorbed molecules, which can vary to a relevant extent for different compound classes. Dąbrowski et al. [23], for example, confirmed that the adsorption of phenols as weak organic electrolytes is much more complex compared to simple porosity effects. For instance, oxidative pretreatment of activated carbon can increase the adsorption of phenolic compounds [50], while acidic oxygen surface complexes decrease the chemisorption of phenols [24,51,52]. The divergent effectiveness of phenol reduction may thus be caused by the different properties of the kraft and soda lignin, respectively. Especially the acid content, pH value or oxygen availability may affect the adsorption capacity of the activated carbon [50]. Specifically, the soda process operates under stronger oxidation conditions, leading to higher contents of carboxylic acids in soda lignins [5,6,20], which might explain the reduced adsorption of phenols in the soda blend AC. Hence, particular attention has to be paid to the characteristics of the raw material lignin when aiming at odor reduction with activated carbon.

#### 4.3.2. Stripping Agent

In contrast to the activated carbon, the influence of the applied processing additive containing polymeric, surface-active substances (‘stripping agent’) on the removal of odorants was more heterogeneous.

In detail, neither the overall odor nor single odor characteristics were significantly reduced in case of kraft blend SA. Analyses of odorants on a molecular level revealed that the majority of phenols, aldehydes, furans and 2-cyclopenten-1-ones were either not reduced or only negligibly reduced. The same was observed in case of the numerically dominating sulfur compounds. Several highly volatile sulfur compounds (RI < 1100) showed even higher OD-factors in the kraft blend SA compared to the untreated blend (No. 2, 3, 4, 6, Table 3). The lack of impact on the load, especially with sulfur-containing odorants, corresponds with the strong odor of the kraft blend SA that was equal to the untreated blend. Accordingly, the stripping agent was ineffective for odor reduction in case of the kraft HDPE-lignin blend under the here applied processing conditions.

With regard to the soda blend, however, odorants were generally reduced by one or, in a few cases, up to two OD-steps, namely for the aldehydes, 2-cyclopenten-1-ones, furan and phenolic compounds. Especially (*E,Z*)-2,6-nonadienal, which was characteristic for the soda blends, was reduced even more from OD-factor 81 to 3. This reduction of the majority of odorant classes reflected the significantly reduced overall odor of 26%. Moreover, since the reduction potential of the stripping agent was found to be similar for single odorant classes, no shift in the single qualities of the odor profile was detectable.

In summary, the stripping agent appeared to be more selective with regard to the chosen material, having a moderate effect only on the soda blend SA. One reason is surely the heavy load of the kraft blends with sulfur compounds originating from the shear- and heat-intensive extrusion process. In general, the removal principle of stripping agents begins with the transport (diffusion) of VOCs to and across the polymer/vapor interface [53]. This is enhanced by the stripping agent generating bubbles and therefore increasing the free volume in the polymer [54]. The consequence is an increased diffusion of volatiles to the gas phase, while final removal occurs via degassing at the end of the extruder. Rate-controlling for this process is the diffusion of target molecules [53,55]. As sulfur compounds are primarily neo-formed during the processing, their removal by the applied stripping agent might be hindered, especially when they are neo-formed in late stages of the extrusion, and bubble-generating effects potentially already lessened. Stripping agents might therefore generally be less effective for lignins resulting from sulfurous isolations processes, such as the kraft process.

To conclude, the above-described approaches led to an initial understanding of the effectiveness of the applied stripping agent and activated carbon on the odor reduction of HDPE-lignin blends. However, further research is of prime importance, for example, by targeting the chemical and functional composition of the applied additives to elucidate their working principle. Especially in the case of the activated carbon, different textural characteristics of different types of activated carbon could also be an influencing factor. For further promotion of odor reduction within the production process of such blends, the elucidation of such mechanisms is prerequisite. Eventually, only the determination of optimal additives (or combinations of additives) and their ideal operating conditions can pave the way towards complete deodorization of lignin blends.

## 5. Conclusions

Neutral and inoffensive odors define one key quality criterion for natural polymers. Especially high odor loads occur in lignin, which hinders high quality applications of equally odorous HDPE-lignin blends. The odor reduction of such blends is challenging and has hitherto been most often addressed within the raw material lignin rather than focusing on the deodorization of the resulting blend. However, this is of prime importance since conditions during the blend production can lead to additional odor formation.

As confirmed in this study, high melt temperatures during heat- and shear-force intense extrusion led to the neo-formation of a variety of particularly odorous sulfur compounds. Hence, resulting *burnt* and *sulfurous* odor sensations were characteristic for the analyzed HDPE-lignin blends, especially in the case of the kraft blends, since remaining sulfurous impurities of the kraft process possibly served as odorant precursors. In the blends with soda lignin, sulfur compounds were less pronounced, but a range of other odorants were detected, among them aldehydes, 2-cyclopenten-1-ones as well as furan- and phenolic compounds. The latter were identified as main odorants in both soda and kraft blends.

In terms of odor reduction, activated carbon was used as additive during the extrusion, which led to a significant positive effect for both HDPE-lignin blends. With 48%, the reduction in odor was particularly powerful in the case of the kraft blend attributable to the substantial decrease from 31 to 18 perceivable sulfur compounds. Regarding phenol reduction, a higher effectiveness was observed for the kraft blend compared to the soda blend, which was linked to different material properties of the lignins.

The second additive tested for potential odor reduction was a commercially available stripping agent with polymeric, surface-active substances. In case of the soda blend, odorants were generally reduced by one to two OD-steps leading to a decrease of 26% in the overall odor. In contrast to that, the effect of the applied stripping agent was less effective for sulfurous odorants that were neo-formed during the extrusion process and, therefore, appears to be less effective for kraft HDPE-lignin blends in general.

In conclusion, odor control proved to be essential during the blend production, and was especially effective in the case of kraft HDPE-lignin blends via the addition of activated carbon. The knowledge gained in this study builds the foundation to further optimize odor of lignin blends, especially for higher quality applications, and offers, in the long run, a high transfer potential with respect to other wood-based material systems.

## Figures and Tables

**Table 1:** Materials and corresponding properties used for the production of the HDPE-lignin blends.

Material	Trade Name	Provider	Material Specifications According to Provider
HDPE	HDPE M80064	Sabic	HDPE injection molding grade with narrow molecular weight distribution; melt flow rate = 8.0 g/10 min
compatibilizer	Fusabond E-MB 100D	DuPont	maleic anhydride grafted HDPE; melt flow rate = 2.0 g/10 min
kraft lignin	BioPiva 100	UPM Biochemicals	kraft softwood lignin from different softwoods; sulfur content <3%
soda lignin	Protobind 1000	PLT Innovations	lignin from agricultural fibrous feedstocks (wheat straw/Sarkanda grass); sulfur free (>90%)
activated carbon	Norit D Ultra	Cabot Norit	powdered activated carbon; total surface area of 1050 m^2^/g; particle sizes of D_10_: 7.4 μm; D_50_: 34 μm; D_90_: 110 μm; apparent density of 500 kg/m^3^; ash content of 11 mass-%; chloride 0.001 mass-%; alkaline pH; methylene blue adsorption min. 20 g/100 g; moisture max. 10 mass-%; filtration time max. 12 min
stripping agent	BYK-P 4200	BYK-Chemie GmbH	processing additive for PE/PP for reduction of odor and VOC; aqueous solution of polymeric, surface-active substances adsorbed onto a PP carrier

**Table 2:** Technical parameters of the production of masterbatches and HDPE-lignin blends.

Parameter	Master- Batch 1	Master- Batch 2	Kraft HDPE-Lignin Blend	Kraft HDPE-Lignin Blend with Activated Carbon	Kraft HDPE-Lignin Blend with Stripping Agent	Soda HDPE-Lignin Blend	Soda HDPE-Lignin Blend with Activated Carbon	Soda HDPE-Lignin Blend with Stripping Agent
abbreviation	MB 1	MB 2		kraft blends			soda blends	
			untreated kraft blend	kraft blend AC	kraft blend SA	untreated soda blend	soda blend AC	soda blend SA
twin screw [rpm]	300	300	400	400	400	400	400	400
dosing rate [kg/h] doser A	4.5	2.5	1.26	1.21	1.26	1.26	1.21	1.26
dosing rate [kg/h] doser B	-	-	0.54	0.61	0.54	0.54	0.61	0.54
temperature profile of barrel segments:								
zone 1 [°C]	180	130	130	130	130	130	130	130
zone 2 [°C]	190	160	170	170	170	170	170	170
zone 3 [°C]	190	180	170	170	170	170	170	170
zone 4 [°C]	190	180	170	170	170	170	170	170
zone 5 [°C]	190	180	190	190	190	190	190	190
zone 6 [°C]	180	180	210	210	210	210	210	210
zone 7 [°C]	170	180	250	250	250	250	250	250
zone 8 [°C]	170	175	230	230	230	230	230	230
zone 9 [°C]	160	170	200	200	200	200	200	200
nozzle [°C]	180	170	200	200	200	200	200	200
melt temperature [°C]	180	180	208	208	208	208	208	208
melt pressure [bar]	10	3	1	3	1	1	1	1
pressure zone 9 [mbar]	-	-	8	8	8	8	8	8

**Table 3:** Detected odorants in untreated and treated (activated carbon/stripping agent) kraft and soda HDPE-lignin blends and respective OD-factors determined via OEDA.

No. ^a^	Odorant ^b^	Odor Quality ^c^	RI ^d^		OD ^e^					
			DB-FFAP	DB-5	Kraft HDPE-Lignin Blend			Soda HDPE-Lignin Blend		
					No Additive	Activated Carbon	Stripping Agent	No Additive	Activated Carbon	Stripping Agent
1	2,3-butanedione	butter-like	984	601	<1	3	<1	1	1	1
2	unknown	blackcurrant-like	1004	n.d.^g^	<1	<1	3	<1	<1	<1
3	thiophene	onion-like, sulfurous	1010	667	1	1	3	<1	<1	<1
4	unknown	blackcurrant-like	1046	824	1	<1	3	<1	<1	<1
5	dimethyl disulfide	cabbage-like	1077	756	3	<1	<1	<1	<1	<1
6	2-methyl-3-hexanethiol ^f^	burned	1109	937	3	1	9	<1	<1	<1
7	1-(methylthio)pentane ^f^	garlic-like, sulfurous	1116	917	81	3	27	1	1	3
8	unknown	sulfurous	1181	n.d.^g^	1	<1	<1	<1	<1	<1
9	unknown	sulfurous	1197	n.d.^g^	1	<1	1	<1	<1	<1
10	4-methoxy-2-methyl-2-butanethiol ^f^	blackcurrant-like	1206	925	27	27	27	<1	<1	<1
11	unknown	sulfurous	1225	n.d.^g^	9	<1	3	<1	<1	<1
12	unknown	sulfurous	1239	817	9	<1	9	<1	<1	<1
13	bis(methylthio)methane	sulfurous, garlic-like	1271	898	81	9	27	<1	<1	<1
14	octanal	citrus-like, soapy	1281	1002	<1	<1	<1	1	<1	<1
15	1-octen-3-one ^f^	mushroom-like	1292	979	1	<1	<1	1	<1	<1
16	2-methyl-3-furanthiol ^f^	broth-like	1304	870	81	27	81	<1	<1	<1
17	1-methoxy-3-methyl-3-pentanethiol ^f^	blackcurrant-like, sulfurous	1324	1036	27	1	9	<1	<1	<1
18	dimethyl trisulfide	garlic-like, cabbage-like	1365	970	729	81	729	81	9	27
19	4-mercapto-4-methyl-2-pentanone ^f^	blackcurrant-like, sulfurous	1374	943	27	<1	243	1	<1	<1
20	1,1-bis(ethylthio)ethane ^f^	sulfurous, burnt	1387	1082	27	9	27	<1	<1	<1
21	3,4-dimethyl-2-pentylfuran ^f^	anise-like, fatty	1413	1203	<1	<1	<1	9	<1	1
22	2-furfurylthiol (2-furanmethanethiol)	roasted coffee bean-like	1428	914	243	27	243	243	27	81
23	acetic acid	vinegar-like	1445	619	1	<1	1	<1	<1	<1
24	methional (3-(methylthio)-propanal) ^f^	cooked potato-like	1446	905	3	<1	1	<1	<1	<1
25	1,2-bis(methylthio)ethane	mushroom-like	1472	1030	3	<1	1	<1	<1	<1
26	2-((methylthio)methyl)furan	cabbage-like	1488	1011	9	1	9	<1	<1	<1
27	(*Z*)-2-nonenal ^f^	green, fatty	1493	1145	3	3	3	9	3	3
28	(*E*)-2-nonenal	fatty, cardboard-like	1523	1160	81	3	27	81	9	27
29	5-methylfurfural	flowery, caramel-like	1564	957	3	<1	3	3	<1	<1
30	(*E,Z*)-2,6-nonadienal	cucumber-like	1573	1159	<1	<1	<1	81	9	3
31	unknown	sulfurous	1579	n.d.^g^	9	3	9	<1	<1	<1
32	unknown	sulfurous	1604	n.d.^g^	3	<1	3	<1	<1	<1
33	3-mercapto-2-methylbutyl acetate ^f^	burnt	1612	1137	27	3	27	<1	<1	<1
34	3-(methylthio)thiophene	cress-like, cabbage-like	1622	1091	243	9	81	1	1	1
35	phenylacetaldehyde	honey-like, flowery	1638	1050	<1	<1	<1	1	1	<1
36	2-methyl-3-(methyldithio)furan	broth-like, meat-like	1667	1178	27	27	27	9	9	3
37	1-mercapto-3-hexanyl acetate ^f^	sulfurous, leek-like	1686	1231	27	1	9	<1	<1	<1
38	unknown	coriander-like	1689	1290	<1	<1	<1	3	1	3
39	(*E,E*)-2,4-nonadienal^f^	fatty	1692	1212	1	<1	1	3	1	1
40	unknown	sulfurous	1712	n.d.^g^	1	<1	<1	<1	<1	<1
41	unknown	sulfurous	1723	n.d.^g^	9	1	1	<1	<1	<1
42	dimethyl tetrasulfide ^f^	sulfurous, cabbage-like	1738	1223	<1	<1	1	<1	<1	<1
43	(*E*)-2-undecenal	coriander-like	1744	1365	<1	3	<1	1	1	<1
44	2-acetyl-2-thiazoline ^f^	roasty, popcorn-like	1750	1107	3	1	3	<1	<1	1
45	3-ethyl-2-hydroxy-2-cyclopenten-1-one	caramel-like	1792	1053	3	3	3	<1	<1	<1
46	cycloten (2-hydroxy-3-methyl-2-cyclopenten-1-one)	lovage-like	1827	1029	9	9	9	3	1	1
47	2-hydroxy-5-ethyl-5-methyl-2-cyclopenten-1-one	caramel-like	1850	1142	9	9	9	3	1	<1
48	guaiacol (2-methoxyphenol)	smoky, smoked ham-like	1862	1087	≥2187	243	≥2187	243	243	81
49	unknown	flowery	1892	1166	3	<1	<1	9	3	3
50	unknown	lovage-like	1929	1181	3	<1	1	1	<1	<1
51	2-methoxy-5-methylphenol	smoky, clove-like	1935	1191	27	3	27	9	3	3
52	unknown	broth-like, meat-like	1963	1403	243	81	243	3	1	3
53	furaneol (4-hydroxy-2,5-dimethyl-3(2*H*)-furanone)	caramel-like	2022	1076	243	27	81	243	27	27
54	*p*-cresol (4-methylphenol)	horse stable-like, fecal	2078	1068	1	1	1	1	1	<1
55	unknown	green, geranium-like	2100	1388	<1	<1	<1	9	3	3
56	2-methoxy-4-propylphenol	phenolic, clove-like	2111	1375	27	3	3	<1	<1	<1
57	2,6-dichlorophenol ^f^	plaster-like, medical	2114	1212	<1	<1	<1	3	3	3
58	2-methoxy-3-vinylphenol ^f^	smoky, clove-like	2123	1240	1	<1	<1	<1	<1	<1
59	eugenol (4-allyl-2-methoxyphenol)	clove-like	2165	1360	81	3	9	81	81	27
60	4-ethylphenol	fecal, phenolic	2169	1171	<1	<1	<1	1	1	1
61	2-methoxy-4-vinylphenol	smoky, clove-like	2182	1317	243	27	243	729	729	243
62	wine lactone ^f^	coconut-like, dill-like	2213	1422	9	1	9	9	3	9
63	*γ*-undecalactone	peach-like	2250	1581	9	3	3	9	9	9
64	2,6-dimethoxyphenol	smoked ham-like, smoky	2260	1363	<1	<1	<1	9	9	9
65	isoeugenol (2-methoxy-4-(1-(*E*)-propenyl)phenol)	smoky, clove-like	2345	1461	729	27	243	27	9	3
66	*γ*-dodecalactone	peach-like	2374	1679	1	1	1	1	1	<1
67	unknown	phenolic, smoky	2425	1446	9	3	9	9	9	3
68	unknown	phenolic, smoky	2450	1495	243	81	243	27	27	9
69	vanillin (4-hydroxy-3-methoxybenzaldehyde)	vanilla-like	2563	1400	≥2187	243	≥2187	≥2187	729	729
70	3-phenylpropanoic acid	honey-like, flowery	2626	1339	<1	<1	<1	243	243	81

^a^ Consecutive order according to elution on DB-FFAP. ^b^ Identification via RI on both capillary columns, odor quality and mass spectral data in comparison to reference compounds (if available) or the NIST database. ^c^ Perceived odor quality at the odor detection port. ^d^ Retention indices (RI) on capillary columns DB-FFAP and DB-5 [34]. ^e^ Odor dilution (OD) factor on DB-FFAP [32]. ^f^ Mass spectrum could not be obtained; identification was based on the remaining criteria given in footnote b. ^g^ n.d.—not detected.

## Data Availability

The data presented in this study are available on request from the corresponding author.

## Appendix A

### Appendix A.1. Reference Compounds and Chemicals

For purification reasons, solvents were distilled prior to use (pentane, dichloromethane obtained from Th. Geyer GmbH und Co. KG, Renningen, Germany). Liquid nitrogen was purchased from Linde Gas (Pullach im Isartal, Germany) and linear alkanes (C_6_–C_26_) from Fluka and Sigma-Aldrich, both Steinheim, Germany.

For the identification of odorants, the following reference compounds were purchased from Sigma-Aldrich (Steinheim, Germany): acetic acid ≥ 99, 2,6-dichlorophenol ≥ 99%, 2,6-dimethoxyphenol ≥ 99%, dimethyl trisulfide ≥ 98%, 2-furfurylthiol ≥ 98%, 2-hydroxy-2,5-dimethyl-3(2H)-furanone (furaneol) ≥ 99%, 2-hydroxy-3-methyl-2-cyclopenten-1-one (cycloten) ≥ 98%, 2-methoxyphenol (guaiacol) ≥ 99%, 2-methoxy-4-vinylphenol ≥ 98, 2-methyl-3-furanthiol ≥ 95%, 4-methylphenol (*p*-cresol) ≥ 99%, 3-(methylthio-)propanal (methional) of unknown purity, (*E,E*)-2,4-nonadienal ≥ 85%, (*E,Z*)-2,6-nonadienal ≥ 95%, (*E*)-2-nonenal ≥ 97%, (*Z*)-2-nonenal ≥ 90%, octanal ≥ 99%, 1-octen-3-one ≥ 50%, phenylacetaldehyde ≥ 90%, 3-phenylpropanoic acid ≥ 99%, *γ*-undecalactone ≥ 98% and (*E*)-2-undecenal ≥ 90%.

4-allyl-2-methoxyphenol (eugenol) ≥ 99%, 2,3-butanedione ≥ 99%, dimethyl disulfide ≥ 98% and 2-methoxy-4-propenylphenol (isoeugenol) ≥ 98% were obtained from Fluka (Steinheim, Germany). Other reference compounds were: (3S,3aS,7aR)/(3R,3aR,7aS)-3a,4,5-7a-tetrahydro-3,6-dimethylbenzofuran-2(3H)-one (wine lactone) ≥ 98% from aromaLAB AG, Freising, Germany), *γ*-dodecalactone ≥ 97% and 2-methoxy-4-propylphenol ≥ 99% from SAFC (Steinheim, Germany), 4-methoxy-2-methyl-2-butanethiol of unknown purity from Takasago (Zülpich, Germany), bis(methylthio)methane ≥ 99% from Alfa Aesar (Kandel, Germany), 4-hydroxy-3-methoxybenzaldehyde (vanillin) ≥ 99%, 4-mercapto-4-methyl-2-pentanone ≥ 98% and 2-methoxy-5-methylphenol of unknown purity from ABCR (Karlsruhe, Germany), 2-acetyl-2-thiazoline ≥ 98% from Chemos (Altdorf, Germany), 3-ethyl-2-hydroxy-2-cyclopenten-1-one ≥ 97% from TCI (Eschborn, Germany), 4-ethylphenol ≥ 99% from Riedel-de-Haen (Seelze, Germany). 2-methoxy-3-vinylphenol was synthesized by members of the work group.

### Appendix A.2. Formulation and Preparation of Masterbatches and Blends

**Table A1:** Formulation of masterbatches and HDPE-lignin blends.

**Material**	**Master-Batch 1**	**Master-Batch 2**	**Kraft** **HDPE-Lignin Blend**	**Kraft** **HDPE-Lignin Blend with** **Activated Carbon**	**Kraft** **HDPE-Lignin Blend with** **Stripping Agent**	**Soda** **HDPE-Lignin Blend**	**Soda** **HDPE-Lignin Blend with** **Activated Carbon**	**Soda** **HDPE-Lignin Blend with** **Stripping Agent**
abbreviation	MB 1	MB 2	kraft blends			soda blends		
			untreated kraft blend	kraft blend AC	kraft blend SA	untreated soda blend	soda blend AC	soda blend SA
HDPE	90	88.5						
compatibilizer	10	9.5						
stripping agent	-	2						
activated carbon			-	5	-	-	5	-
kraft lignin			30	29	30	-	-	-
soda lignin			-	-	-	30	29	30
MB 1			70	66	35	70	66	35
MB 2			-	-	35	-	-	35
